# In Vitro Protein Digestibility and Fatty Acid Profile of Commercial Plant-Based Milk Alternatives

**DOI:** 10.3390/foods9121784

**Published:** 2020-12-01

**Authors:** Eliana Martínez-Padilla, Kexin Li, Heidi Blok Frandsen, Marcel Skejovic Joehnke, Einar Vargas-Bello-Pérez, Iben Lykke Petersen

**Affiliations:** 1 Department of Food Science, University of Copenhagen, Rolighedsvej 26, DK-1958 Frederiksberg C, Denmark; elimarp23@gmail.com (E.M.-P.); kexin35874@163.com (K.L.); heidi.frandsen@siccadania.com (H.B.F.); marcel@food.ku.dk (M.S.J.); 2 SiccaDania, Pilehøj 18, DK-3460 Birkerød, Denmark; 3 Department of Veterinary and Animal Sciences, University of Copenhagen, Grønnegårdsvej 3, DK-1870 Frederiksberg C, Denmark; evargasb@sund.ku.dk

**Keywords:** milk substitutes, non-dairy, almond drink, oat drink, soy drink, hemp drink, nutritional value

## Abstract

Plant-based milk alternatives (PBMA) are a new popular food trend among consumers in Europe and North America. The forecast shows that PBMA will double their value by 2023. The objective of this study was to analyze the nutritional value of commercial products in terms of their fatty acid profile and protein digestibility from commercial PBMA. Eight commercially available PBMA were selected for fatty acid analysis, performed with gas chromatography of methylated fatty acids (GC-FAME), and, from these, four commercial products (almond drink, hemp drink, oat drink, and soy drink) were selected for a short-term in vitro protein digestibility (IVPD) analysis. The fatty acid analysis results showed that most of the products predominantly contained oleic acid (C18:1 ω-9) and linoleic acid (C18:2 ω-6). Hemp drink contained the highest omega-6/omega-3 (ω6/ω3) ratio among all tested products (3.43). Oat drink and almond drink were the PBMA with the highest short-term protein digestibility, non-significantly different from cow’s milk, while soy drink showed the lowest value of protein digestibility. In conclusion, PBMA showed a significant variability depending on the plant source, both in terms of fatty acid composition and protein digestibility. These results provide more in-depth nutritional information, for future product development, and for consumer’s choice.

## 1. Introduction

Over the last few years, plant-based milk alternatives (PBMA) have become one of the most popular food trends among consumers from North America and Europe [1]. In 2017, the worldwide value of PBMA was approximately 9 billion U.S. dollars, and the forecast shows that their worth will double by 2023 [2]. Originally, these products were mainly directed for a segmented market, consumed by people with lactose intolerance or other nutritional restriction, such as cow’s milk protein allergy [3]. Today, PBMA act as a direct substitute to cow’s milk for the mass market, claiming to be a healthier and more sustainable option for consumers [4,5,6,7]. They are an attractive food product for consumers who wish to lower or avoid the consumption of animal products [4,8,9]. Since the 1970s, high consumption of food products from animal origin has been related to greater risk of cardiovascular diseases (CVD) due to high content of saturated fats and cholesterol [10,11,12], and an increased intake of fruits and vegetables has been associated inversely to the risk of CVD, and other chronic diseases [13]. In addition, the scientific evidence on the environmental consequences of the animal food industry has led to a new concern for consumers: to eat products that do not harm the environment and are sustainable [14,15]. This has posed a negative view to the dairy industry since cows have shown to be the major contributor for greenhouse gas (GHG) emissions compared to other farming animals [16], although livestock production is only responsible for 18% of all GHG emissions [17].

The PBMA are produced by the extraction of a given plant source (cereal/legume/seed/pseudocereal) with water, followed by a removal of the sediment from the liquid [18]. Ingredients, such as oil, sugar, flavoring, vitamins, minerals, and stabilizers, are added to the fluid extracted, and then the mix is homogenized to a particle size that mimics cow’s milk appearance, followed by pasteurization or sterilization heat treatment [18]. Merely for the plant-extraction stage, several operations need to be performed like grinding, soaking, use of enzymes, increasing pH, and high temperatures [18]. Despite being highly processed products, consumers of PBMA perceive them as a healthy product [7,9,19,20]. Concerning the nutritional value, PBMA are often promoted as being free of cholesterol and low in saturated fats in contrast to cow’s milk [21]. On the other hand, the protein content of PBMA remains at minimal levels, soy drink being an exception having a protein content comparable to cow’s milk [22,23].

However, the nutritional evaluation concerning these products has not been extensively investigated beyond the content from the nutrient declaration [19,23]. These products claim not to be cow’s milk but act as immediate substitutes, which can create confusion among consumers, especially in regard to the nutritional value that will depend on the plant source and manufacturing process [19,21]. Concerning cow’s milk, the presence of saturated fatty acids (SFA) has been a determinant factor for health conscious consumers to consume low fat milk [24,25], although a few recent studies have shown that SFA from dairy consumption were not associated with higher risk of cardiovascular diseases [26,27,28]. Many of the PBMA are very low in fat content; however, often, vegetable oils, like sunflower oil, are added to mimic the smooth mouthfeel of cow’s milk [9], which alters the final fatty acid (FA) profile of PBMA [18], and this has not been investigated previously.

A positive nutritional attribute of cow’s milk is its protein content. The consumption of cow’s milk has been positively associated with the increase of protein body levels among children through numerous interventional studies [29,30]. Conversely, the exclusive consumption of rice drink (250 mL/day) in toddlers and children demonstrated a poor weight gain and lower height due to very low protein quantity [31,32,33]. Although soy drink has been a comparable product to cow’s milk in protein content, soy beans contain trypsin inhibitors, antinutrients known to limit the activity of digestive enzymes trypsin and chymotrypsin, thereby reducing the full absorption of the ingested proteins in the small intestine [31,32,34]. Nevertheless, heat treatment or high-pressure processing usually reduces the activity of these antinutrients [35,36] but to which extent varies depending on the manufacturing process [37,38]. Recent food product developments are aiming for a higher protein content in PBMA, due to consumers preference for higher protein food products [9,39,40,41], or using new protein sources, such as hemp seed [42,43]. Apart from providing protein, cow’s milk is also a good source of calcium, and, because of this, many PBMA are fortified with similar calcium amounts as cow’s milk [7,18,22]. However, the calcium absorption in PBMA can be altered due to the presence of antinutrients, such as phytates or oxalates, thereby decreasing the total calcium absorbed [44].

Another important aspect to take into account when developing new PBMA’s is the protein quality, which is mainly determined by the amino acid composition, the protein digestibility, and the amino acid bioavailability [45]. Particularly, the level of indispensable amino acids (IAAs) is a major factor when assessing the quality of a dietary protein source [45]. Plant-based protein sources like cereals (e.g., millet, rice, and oats) are generally lower in IAAs, such as methionine, lysine, tryptophan, and threonine, compared to animal-based protein sources (e.g., cow’s milk) [46]. Furthermore, plant-based protein sources are usually also less digestible than animal-based protein sources [47]. Dietary protein quality can be assessed under in vivo conditions by measuring the true ileal digestibility of IAAs using the DIAAS (Digestible Indispensable Amino Acid Score) method [48]. Using this approach, it has been shown that cow’s milk has higher DIAAS value than soy drink [49]. A study evaluating the protein quality of oats showed a DIAAS below the recommended value for daily intake; therefore, it was suggested to complement oats with other higher quality protein sources [50]. This is another aspect to take into account when developing new plant-based milk alternatives.

The aim of this study was to investigate commercially available PBMA, with a focus on their fatty acid profiles, as well as in vitro protein digestibility (IVPD), to obtain a more detailed view of the nutritional values of different PBMA in comparison to cow’s milk. The fatty acid profile of different commercial products (almond drink, coconut drink, hazelnut drink, hemp drink, oat drink, quinoa drink, and soy drink) was evaluated through gas chromatography (GC) analysis, and the IVPD of selected products (almond drink, oat drink, soy drink, and hemp drink) was determined in comparison to cow’s milk. This study provides more in-depth information for future product developments, further nutritional research, and information for consumers about the nutritional value of PBMA, thereby providing a sound base for consumers’ personal choice regarding their diet.

## 2. Materials and Methods 

### 2.1. Samples

Samples were collected from eight commercially available PBMA in the city of Copenhagen, Denmark during the months of February and March 2019 (*n* = 8): almond drink (*Ecomil*, 2.1% fat), coconut drink (*Naturli*, 0.9% fat), hazelnut drink (*Alpro*, 1.6% fat), hemp drink (*Ecomil*, 2.9% fat), oat drink (*Oatly organic*, 0.5% fat), quinoa drink (*Ecomil*, 1.2% fat), rice drink (*Naturli*, 1.1% fat), and soy drink (*Naturli*, 2.1% fat), as well as whole milk (*Arla*, 3.5% protein). An aliquot of 50 mL was taken from each of these drinks, frozen, and freeze-dried (Gamma 1–16; LSCPlus, Christ, DE) prior to analysis. 

### 2.2. Chemicals and Reagents

Chloroform, cyclohexane, fatty acid internal standards (hexanoic acid, decanoic acid, and heptadecanoic acid), boron trifluoride, pepsin from porcine gastric mucosa (P7000, 920 units/mg protein), pancreatin from porcine pancreas (P1750, 4× USP), sodium cholate hydrate from ox or sheep bile (purity ≥ 99%), acetic acid (purity ≥ 99.8%), and picryl sulfonic acid solution 5% (*w*/*v*) in H_2_O were purchased from Sigma Aldrich Denmark A/S (Copenhagen, Denmark). The concentration of the total internal standard solution was 10 mg∙mL^−1^ of fatty acids in hexane. Methanol and sodium hydroxide were purchased from VWR Chemicals (Stříbrná Skalice, Czech Republic). Sodium hydrogen carbonate and di-sodium tetraborate-decahydrate were purchased from Merck (Darmstadt, Germany). Ultrapure water obtained from Milli-Q Plus system (Millipore Corporation, Milford, MA, USA) was used for all buffers and reagent preparations.

### 2.3. Fatty Acid Analysis

The fatty acid composition of the plant lipids was based on the conversion into fatty acid methyl esters (FAME) followed by analysis with GC-Flame ionization detection (FID) (Agilent Technologies GC System 7820A, Santa Clara, CA, USA). Lipids were extracted from the freeze-dried samples with chloroform-methanol 2:1 (*v*/*v*). The fatty acid methyl esterification procedure was according to the AOAC official method no 969.33 with minor modifications. Extracted plant lipids (20 mg) were added to methanolic NaOH (0.5 M, 1 mL), and internal standard solution (hexanoic acid, decanoic acid, and heptadecanoic acid, 10 mg∙mL^−1^, 200 µL), and vortex mixed with boron trifluoride (BF3, 1.5 mL) until there were no visible solids inside. The mixture was heated for 2 min at 70 °C and then transferred to a 5 mL volumetric flask. Cyclohexane (1 mL) and saturated NaCl solution were added. The mixture was separated in two phases after standing and the cyclohexane phase was transferred to GC vials. For the GC analysis, a fused silica capillary column SP^TM^ 2370 (30 m × 200 µm, ID 25 nm) from Supelco (Bellefonte, PA, USA) with helium as the carrier gas at a flowrate of 2.477 mL∙min^−1^ was used. The oven temperature program was: 60 °C for 2 min, 10 °C∙min^−1^ to 200 °C, 5 °C∙min^−1^ to 240 °C and hold at 240 °C for 7 min. The temperature of the flame ionization detector was 250 °C. An injection with a split ratio of 52.735:1 was used. Fatty acids were identified using FA methyl ester (FAME) standard 37 Component FAME mix; Supelco (Bellafonte, PA, USA).

### 2.4. In Vitro Protein Digestibility (IVPD)

Sample selection criteria for the protein digestibility assay was a protein content of ≥1 g/100 mL (declared in the nutritional label) from the previous analyzed PBMA samples. Products containing less than 1g protein/100 mL were not included, due to analytical accuracy, precision, resolution, and sensitivity with the IVPD methodology. This resulted in the selection of almond drink (*Ecomil*, 1% protein), hemp drink (*Ecomil*, 1% protein), oat drink (*Oatly organic*, 1% protein), and soy drink (*Naturli*, 3.7% protein). Whole milk (*Arla*, 3.5% protein) was included in the analysis for comparison. Free alanine amino acid (Merck, Darmstadt, Germany) was included as standard reference sample representing 100% protein digestibility during each stage of digestion. Blank samples containing only buffer and enzymes were included to correct for enzymatic self-digestion. Bovine serum albumin (BSA) (Sigma-Aldrich, Copenhagen, DK) was included as a positive control due to its relatively high protein digestibility [51]. The nitrogen content of the samples was determined from the freeze-dried samples with the Dumas combustion method using an Elementar Vario Macro Tube CHNS analyzer (Elementar Analysensysteme GmbH, Hanau, Germany) with helium as carrier gas. The standard conversion factor to obtain the crude protein content from nitrogen content was 6.25 (Table S1).

Gastrointestinal protein digestion was simulated using a multi-step static IVPD model system [52], with a few modifications. Due to relatively low protein contents in the selected PBMAs, all samples were measured to contain 25 mg of protein on dry weight basis. The enzymatic hydrolysis consisted of two digestion stages: gastric digestion with pepsin (1 h, 37 °C, pH 1–2), followed by a short-term intestinal digestion with pancreatin (1 h, 37 °C, pH 7–8). The enzyme: substrate ratios were kept constant during the digestion stages at about 1:25 and 1:5 (*w*/*w*), respectively. Aliquots of samples were withdrawn before digestion (untreated samples), after pepsin digestion, and after pancreatin digestion, and immediately diluted 1:10 (*v*/*v*) in sodium borate buffer (pH 10.0) to inactivate the enzymes. For the quantification of protein digestibility of the samples, a trinitrobenzene sulfonic acid (TNBS)-based assay was conducted in 96-well microtiter plates [52]. The concentration of free α-amino groups in the samples was related to the alanine reference sample, and an alanine standard solution added within each plate to calculate the IVPD (%). The level of hydrolysis of untreated samples was subtracted to obtain corrected IVPD values for the pepsin digestibility (%), pancreatin digestibility (%), and total digestibility (%).

### 2.5. Statistical Analysis

R Studio (library version 3.5.1) software was used for statistical analyses. For the fatty acid profile analysis, mean and standard deviation (SD) (*n* = 3) for each product and each fatty acid was calculated. Test for significant differences between samples was One-way analysis of variance (ANOVA) and Tukey post hoc test that was performed for all PBMA except coconut drink, due to a highly different fatty acid profile from the rest of products, and cow’s milk since the values obtained were from a food database [53]. For the in vitro protein digestibility, mean and standard deviation (*n* = 3) for each product and each digestion stage was calculated. Similarly, ANOVA and Tukey post hoc test were performed among all samples. *p* value <0.05 was considered statistically significant for all experiments.

## 3. Results

A description of the samples (list of ingredients and nutritional declaration), taken from the package labeling, is shown in Table 1.

### 3.1. Fatty Acid Analysis (GC-FAME)

Table 2 shows the fatty acid composition of the eight PBMA determined by GC-FAME. Significant statistical differences were observed between all products (*p* < 0.001). Coconut drink presented a high content of SFA (94% of total lipid content), mainly lauric acid (C12:0) and myristic acid (C14:0), while the rest of the PBMA had values of SFAs ranging from 8% to 23%. Mono-unsaturated fatty acid (MUFA) values varied among the samples, with hazelnut drink having the highest value (80%) and soy drink the lowest (22%). Hemp drink had the greatest source of polyunsaturated fatty acids (PUFA) with a content of 74%. Oleic acid (C18:1) and linoleic acid omega-6 (C18:2, ω-6) were the greatest source of fatty acids for most of the PBMA. Almond drink, hazelnut drink, and quinoa drink had oleic acid (C18:1) as the main source of fatty acids, with 59%, 80%, and 65% of total fatty acids, respectively, all products being significantly different in this fatty acid (*p* < 0.001).

On the other hand, oat drink, soy drink, rice drink, and hemp drink presented higher levels of linoleic acid omega-6 (C18:2, ω-6), with no significant differences between soy drink and rice drink (*p* = 0.9458), as well as between hemp drink and soy drink (*p* = 0.0835). Regarding SFA, palmitic acid (C16:0) contents varied from 6% to 17% content within the different drinks, with similar values for hemp drink and quinoa drink (*p* = 0.9803). The only PBMA that offered a source of α-linolenic acid (C18:3 ω-3) were hemp drink with a content of 16%, followed by soy drink (7%). These results show a highly different profile from cow’s milk, where the principal fatty acids are the saturated fatty acid palmitic acid (32%) and the monounsaturated fatty acid oleic acid (18%) [53]. Cow’s milk presented 30% lower content of SFA than the coconut drink. Concerning the omega 6: omega 3 ratio (ω6/ω3), hemp drink appeared with a ratio of 3.43, similar to cow’s milk (3.43), followed by soy drink with a ratio of 7.52.

### 3.2. In Vitro Protein Digestibility (IVPD)

The samples selected for the in vitro protein digestibility assay were almond drink, hemp drink, oat drink, and soy drink since the rest of PBMA samples had a protein content of less than 1 g/100 mL, as stated in the nutritional declaration (Table 1). Table 3 shows the results from the in vitro protein digestibility analysis of PBMA samples and whole milk sample with TNBS detection, as well as statistical differences among samples for each digestion step.

The in vitro protein digestibility simulating stomach conditions was performed through a 1-h incubation with pepsin. Of all samples, BSA (control) had the significantly highest pepsin digestibility (*p* < 0.001). Cow’s milk showed a similar level of pepsin digestibility as the PBMA based on oats and soybeans. Soy drink was pepsin digested to a significantly higher degree than hemp drink (*p* < 0.01) and almond drink (*p* < 0.05). Following the simulated gastric digestion, the IVPD simulating small intestinal conditions was conducted by a 1-h incubation with a pancreatic enzyme mixture. Similar to the gastric digestion phase, BSA showed the highest value of pancreatic digestibility, which was significantly different from the PBMA (*p* < 0.001) and significantly different from cow’s milk (*p* < 0.01). Soy drink had the lowest level of pancreatic digestibility compared to all other samples (*p* < 0.001), followed by hemp drink. Cow’s milk, almond drink, and oat drink were not significantly different from each other (*p* = 0.56 for cow’s milk vs. almond drink and *p* = 0.05 for almond drink vs. oat drink, respectively), and these were the most easily digested drinks in this digestion phase.

Total pepsin and pancreatin digestion of the PBMA showed that soy drink and hemp drink had the lowest values of total in vitro protein digestibility and that these were non-significantly different from each other (*p* = 0.49). Cow’s milk had a similar total protein digestibility as oat drink (*p* = 0.26) and almond drink (*p* = 0.44). As expected, BSA was the most digestible protein after both stages of digestion, being significantly different from all the other samples (*p* < 0.001).

## 4. Discussion

The objective of the study was to determine the profile of fatty acids and level of in vitro protein digestibility of commercially available PBMA in order to evaluate the differences between them, as well as to compare them to cow’s milk, thereby providing support to build up more nutritional information regarding new commercial PBMA.

### 4.1. PBMA Fatty Acid Profile

The results from the fatty acid profile analysis of PBMA showed that oleic acid (C18:1) and linoleic acid (C18:2, ω-6) were the major fatty acids present in all products, except coconut drink sample. These results were in accordance with Vargas-Bello-Perez (2017) [54], who also found oleic acid (C18:1) and linoleic acid (C18:2, ω-6) to be the dominating fatty acids in PBMA. Vegetable oils are usually added to PBMA that come from plant sources with a minimal fat content to positively increase the sensory properties of these products and achieve a greater consumer acceptance [19]. In this study, three of the PBMA contained added oil: rice drink, quinoa drink, and hemp drink. 

When comparing the FA profile of rice drink with the FA profile of milled rice, the results are different, as expected. Milled rice is higher in saturated fatty acids (24 %), where the amount of oleic acid (C18:1) and linoleic acid (C18:2, ω-6) is 38%, and 35%, respectively [55]. The amount of oil added to the PBMA is unknown, although, when comparing the FA profile of sunflower oil with the FA profile of the rice drink, results are in congruence [56]. Sunflower oil contains 35% oleic acid (C18:1) and 59% linoleic acid (C18:2, ω-3) [56]. In the present study the linoleic acid (C18:2, ω-6) content was 55.6% in the rice drink, indicating that sunflower oil accounts for about 85% of the oil in the rice drink. 

With respect to hemp drink, this PBMA had been added hemp oil, and the FA profile was in congruence with the FA profile of hemp oil [57]. Concerning the quinoa drink, the FA profile differed from the FA profile of quinoa. Quinoa contains a fat content of 2–10%, with a high content of linoleic acid (C18:2, ω-6) (48.1–52.3%) and oleic acid (C18:1) (22.8–29.5%), as well as a smaller proportion of palmitic acid (C16:0) (9.2%) and α-linolenic acid (C18:3, ω-3) (4.6–8.0%) [58]. The quinoa drink was declared to have added sunflower oil, as also was the case in the evaluated PBMA rice drink; however, their FA profiles were significantly different, meaning that probably a different type of sunflower oil was added to the quinoa drink since the analyzed level of α-linolenic acid (C18:3, ω-3) was only 25%. The sunflower oil added was, thus, probably a “high-oleic sunflower oil”, which contains 79% of MUFA [59], similar to the proportion of MUFA found in the quinoa drink.

In regard to oat drink results, these were in accordance with a study on oat cultivars in the Czech Republic [60], where only small differences in the fatty acid profile could be noticed. According to the results from this present study, oat drink contained 5% more of MUFA, 5% more of PUFA, and 8% less of SFA compared to oat cultivars from the Czech Republic. These small differences could be due to location of oat production [61], since the product analyzed contained oats originally from Sweden, and climate and soil conditions are determinant environmental factors for the final product [62]. The analyzed level of SFA in the oat drink corresponded to the declared level (19% vs. 20%).

Compared to the typical levels of FA in almonds, the almond drink investigated in this study had a slightly different fatty acid profile [63], with higher amounts of the SFAs palmitic acid (C16:0), stearic acid (C18:0) and lauric acid (C12:0). Almonds were reported to be rich in oleic acid (C18:1) [63], and this was in agreement with our results, where oleic acid (C18:1) was the major source of fatty acids in the almond drink. When comparing the percentage of SFAs in the almond drink with the nutrient declaration from the package labeling, the percentage of analyzed SFAs corresponded fairly to the amount of fatty acids declared (23% vs. 19%). 

In the case of soy drink, this product was found to have around 7% higher content of palmitic acid (C16:0) compared to the FA profile of commercial soybeans [64]. The oleic acid content in soy drink was similar to the level in soybeans [64]. Linoleic acid (C18:2, ω-6) content was higher in soybeans [64] than in the soy drink evaluated in this present study. Our analyzed level of SFA was, however, 5% lower than the declared level (14% vs. 19% Table 2 and Table 3).

The fatty acid profile of hazelnut drink was in accordance with a study done with 75 different types of hazelnuts from Europe [65], showing a mean of oleic acid (C18:1) content of 81% and 11% for linoleic acid (C18:2, ω-6). 

Coconut drink contained a high content of SFA with lauric acid (C12:0) as the dominant FA (47.20 ± 0.38 %). This FA is a short-chain fatty acid (SFCA), and some researchers have addressed the healthiness of SCFA because they are rapidly metabolized in the liver and have the ability to convert into other metabolites, such as ketone bodies, which provide immediate energy to the brain and heart [66]. Nevertheless, the production of ketone bodies promotes liver endoplasmic reticulum stress since this production had the objective to only appear when fasting [67]. Differently, cow’s milk contained a higher level of SFA palmitic acid, which has been a controversial fatty acid with different nutritional studies relating it to obesity, diabetes type 2, and cancer [68]. However, recently, it has been shown that palmitic acid could increase high-density lipoprotein (HDL) blood cholesterol when replacing carbohydrates in the diet [69]. 

When examining cow’s milk profile, it is essential to remark that cow’s milk FA profile is a more complex food matrix, estimated to contain over 400 different FA [70]. Although cows are fed plant-based, when dietary lipids enter into the rumen, they suffer a process called biohydrogenation, whereby microorganisms add H atoms to saturate the double bonds of dietary unsaturated FA, as they are toxic to cows [70]. Therefore, saturated FA mostly composes cow´s milk and this group of FA can account for more than 70% of FA, as observed in this study. Saturated fats have been related as promotors for unhealthiness among the population; however, a recent meta-analysis showed that replacing saturated fats with polyunsaturated fat makes no significant difference to the prevalence of coronary disease [56]. In addition, SFA are essential components of cell membranes [68]. It is noteworthy mentioning that cow’s milk has a wide variety of fatty acids that could have positive or negative effects on human health. For example, some saturated FA are related to the development of atherosclerosis (i.e., C12:0, C14:0, and C16:0) and coronary thrombosis (i.e., C14:0, C16:0, and C18:0) in humans [71]. On the other hand, bovine milk fat also contains unique trans FA (i.e., C18:1 trans-11) that may prevent the development of cardiovascular disease [72]. One of the most notorious groups of milk FA are those related to the conjugated linoleic acid (CLA), and they are mixture of isomers of octadecadienoic acid containing conjugated double bonds. They occur naturally in dairy products as a result of rumen bacteria, and one of the main forms is C18:2 cis-9,trans-11, which has been related to reduce atherosclerosis, anti-diabetic effect, and improvement of immune function [73]. Additionally, some short-chain FA, such as butyric acid (C4:0), have been reported to have antitumor activity and anti-inflammatory activity [74].

One critically aspect calculated was the omega-6 to omega-3 (ω-6:ω-3) ratio. This was calculated for the PBMA products evaluated in this study (Table 3), and the majority of PBMA products showed an imbalance in this ratio, with the exception of hemp drink, which presented a ω-6:ω-3 ratio of 3.41. Hemp drink contained an omega-3 content of 16%, which was the highest detected among all PBMA analyzed in this study. From Table 1, it can be observed that this product had hemp oil added to hemp drink, and this potentially benefited the final omega-6 to omega-3 (ω-6:ω-3). This counts as a strongly positive factor for selecting this type of PBMA, from a health perspective, since a healthy ratio of omega-3 ratio (ω-6:ω-3) occurs in the ranges from 1:1 to 4:1 [75]. Cow’s milk FA content also presented an omega-6 to omega-3 (ω-6:ω-3) ratio of 3.41; however, the amount of linoleic acid omega-6 (C18:2, ω-6) and α-linolenic acid omega-3 (C18:3, ω-3) were minimal (1.46 % and 0.42%). The optimal dietary intake of omega-3 and omega-6 FA is continuously being debated among scientists [76,77,78]. According to European Food Safety Authority (EFSA), the current guidelines are to ensure an energy intake of 4% and 0.5% from linoleic acid omega-6 (C18:2, ω-6) and α-linolenic acid omega-3 (C18:3, ω-3), respectively [79], while no specific omega-6 to omega-3 ratio (ω-6:ω-3) has been recommended. Nevertheless, studies find that too high levels of omega-6 cause some chronic degenerative diseases, such as cardiovascular disease and obesity [75,80]. Recent studies found an association with imbalanced ω-6:ω-3 ratio, low omega-3 levels and mental health, specifically with mood disorders and depression in adults [81,82]. In the modern diet, omega-6 can be consumed up to 20 times more than omega-3 [80]. The efficiency of α-linolenic acid omega-3 (C18:3, ω-3) bioconversion into longer omega-3 chains is still undergoing investigations [76,77]. However, it is well-known that α-linolenic acid omega-3 (C18:3 ω-3) and linoleic acid omega-6 (C18:2, ω-6) are metabolized in parallel competitive pathways in the eicosanoid metabolism [78], which is one reason to have a deeper look at the ratio of these in our study. 

Currently, there are no long-term studies that have analyzed the impact of omega-6 to omega-3 (ω-6:ω-3) ratio from the consumption of PBMA to the overall health status. However, the amount of lipids is very low in most PBMA (<2 g/100 mL), and, by drinking one glass of PBMA (250 mL), it only contributes to a small percentage of total fat intake in an adult (recommended 44–77 g of fat a day) [83]. A suggestion to improve the omega-6 to omega-3 ratio in PBMA would be changing the added oils that are high in omega 6 fatty acids (sunflower, safflower, soybean oils) to those high in omega-3 (flax, perilla, chia, rapeseed oils) [80].

Additional nutritional indices to assess fatty acids in relation to cardiovascular health are the Index of Atherogenicity (IA) and the Health-Promoting Index (HPI), among others [84]. IA is obtained through the following formula, IA = {C12:0 + (4 × C14:0) + C16:0}/ƩUFA and evaluates the atherogenicity of foods, where a low IA value is considered to be healthy [84]. When applying this formula to the values of PBMA obtained in the present study, with exception of coconut drink, the IA for the PBMA, are all below 0.1, with hazelnut drink, quinoa drink, and hemp drink having the lowest values (≈0.06, ≈0.07, and ≈0.08, respectively). The IA value for the coconut drink was approximately 20.92. Compared to cow’s milk (IA ≈ 3.04), coconut drink has an extremely high value of IA, due to the low quantity of unsaturated fatty acids (UFA) and high quantity of C12:0 and C14:0 (47.2 and 19.37, respectively). These two FA, together with C16:0 contribute to the adhesion of lipids to cells in the circulatory system [85]. Contrary to IA values, where low values are interpreted healthier, high values of HPI are interpreted as healthy [84]. The formula for calculating HPI is the inverse to IA’s formula (HPI = ƩUFA/{C12:0 + (4 × C14:0) + C16:0}) [84]. Interestingly, in this matter, the difference between coconut drink and cow’s milk is relatively low (0.05, and 0.33 respectively). When compared the HPI values of the rest of PBMA, these values ranged from ≈4.72 (oat drink) to ≈15.45 (hazelnut drink). Considering these nutritional indices, hazelnut drink (HPI ≈ 15.45), quinoa drink (HPI ≈ 13.58), and hemp drink (HPI ≈ 13.06) could be considered the most cardiovascular healthy PBMA, and special attention should be considered to coconut drink, according to these indices. 

### 4.2. PBMA In Vitro Protein Digestibility (IVPD)

The purpose of this experiment was to investigate the protein digestibility of different commercial PBMA compared to cow’s milk, and this is the first study to address an IVPD analysis between different plant source PBMA and cow’s milk. Almond drink and oat drink both had a total digestibility being non-significant different to cow’s milk after IVPD analysis, while soy drink had the least significantly digestible protein value. The results regarding BSA protein digestibility from this present study are in accordance with values reported by Joehnke [52], using a nearly similar IVPD methodology, where they found a BSA pepsin digestibility of 7.5 ± 0.2%, pancreatin digestibility of 22.1 ± 1.0%, and total BSA digestibility of 29.6 ± 1.0% [52]. BSA has been included as a reference representing a highly digestible protein source. In the study by Joenhke [52], α-lactoglobulin and β-lactoglobulin were also analyzed, showing similar values (28.6% and 24.6%, respectively) of total digestibility to cow’s milk in this present study. Nevertheless, the digestion conditions in this present study were adjusted, since a protein amount of 25 mg was used for the analysis, whereas Joehnke and co-workers used a protein amount of 50 mg. 

Studies on in vivo and in vitro protein digestibility of almonds have generally shown that their proteins are highly digestible [86,87,88]. However, it is suggested that both almond and oat protein sources have a lower protein quality, when comparing to the protein recommendations of FAO/WHO [86]. Protein quality is defined as the ability of a dietary protein to meet the needs for regular metabolism and maintenance or growth of body tissues [89]. Most limiting amino acids in almonds are methionine, lysine, and threonine [90,91], and this is important for the final protein bioavailability since, if any of the limiting amino acids will be missing, the protein levels after absorption will be lower [89]. A recent study testing different types of almonds from the U.S. found that there are indeed differences between them in relation to IVPD determined by a pH drop method [92]. In the same study, lysine was determined as the limiting amino acid [92]. In relation to oats, some authors have found that the most limiting amino acids were lysine and threonine [51,93], and another study looking at four types of oat cultivars in Brazil determined lysine as the limiting amino acid [94]. In the later study, they investigated the nitrogen balance in vivo in rats evaluating oats from the different cultivars compared to casein, where casein had significantly higher nitrogen level (*p* < 0.05) than oats [94]. The apparent digestibility for casein was around 93.8%, whereas it was around 83% for oats, while other protein quality indicators showed a higher protein quality for casein [94]. To make the proteins in oats more accessible, enzymes and protein-glutaminase can be added to the process, also with the purpose to give a more uniform oil droplet and larger stability [95]. In addition, some food companies will compensate for the low protein content in the oat drinks by using protein supplementation [18].

The low digestibility of soy drink could be explained by the presence of antinutrients, e.g., trypsin inhibitors [96]. In a study where different treatments of soy drinks were evaluated by IVPD, the product that was ultra-high-temperature (UHT)-treated had a significantly higher protein digestibility compared to the sample that was ultra-high pressure homogenized (UHPH) [97]. There are conflicting results between different industrial treatments to soy drinks since a higher trypsin inhibitor activity has been reported in samples treated with UHPH compared to what has been measured in soy drinks treated by UHT [98]. Other authors reported that 90% of the trypsin inhibitors were inactivated under high-pressure processing [35]. The soy drink evaluated in this present study was UHT treated. A recent review by Munekata et al., evaluating the effect of different food processing technologies on the nutritional properties of PBMA, including protein digestibility, recommended to use high pressure and pulse electric fields (PEF) over conventional heat treatment to preserve the properties in PBMA [99]. A recent study investigating different treatment conditions with microwave (100 °C for 8 min) and conventional processing (100 °C for 30 min) showed an increase in the IVPD (87% and 92%, respectively) compared to the IVPD in raw soy drink (80.5%) [100]. The trypsin inhibitor activity (TIA) was also evaluated in the later study, showing that microwave treatment to soy drink reduced TIA to 3% and conventional treatment to 1%, whereas raw soy drink had a TIA of 8% [100]. The type of food processing is critical for the final nutritional quality, and this needs to be considered when developing new PBMA products. 

There is a vast variety of in vitro protein digestibility methodologies, which makes it difficult to compare results. Another factor adding to the difficulty of comparing results is that the majority of samples discussed in relation to the in vitro experiments are produced under laboratory conditions, as opposed to the industrially produced commercial PBMA. The objective of this study was to qualify the protein content of the most popular available drinks on the Danish market, by giving an indication of the amount of protein available for metabolism, as measured by IVPD. However, information of how the products were processed industrially by each company was limited to only the regulated obligatory information according to Regulation (EU) No 1169/2011. A further step would be to do an interventional study with current commercial plant-based drinks versus cow’s milk. Some interventional studies have been made comparing the protein intake from some of the plant-based milk alternatives to cow’s milk, with outcomes for plant-based milk alternatives consumers of lower growth in childhood, and levels of protein below the recommendations for them [33,101]. An option to increase the protein quality of plant-based milk alternatives could be to combine the plant-sources, legumes with grains, or nuts and seeds, to complement their amino acid profile [102,103]. This is a first exploratory study that obtained more insights from the protein functionality (in vitro protein digestibility) perspective on commercial manufactured plant-based milk alternatives in northern European countries, such as Denmark.

## 5. Conclusions

Overall, commercially available plant-based milk alternatives showed a significant variability depending on the plant source, both in terms of fatty acids and in vitro protein digestibility, similarly when compared to cow’s milk. Hemp-drink was found as the product with the highest nutritional value with respect to the fatty acids, due to the ratio of omega 6 to omega 3 of 3.41 being within the recommended ratio (1:1 to 4:1) for a healthy diet. With respect to the in vitro protein digestibility, the PBMA showing the lowest digestibility was soy drink, whereas oat-drink, almond-drink, and cow’s milk showed significantly better digestibility. These results provide insights for future product developments and further nutritional research that can provide to consumers more information for their future choices.

## Figures and Tables

**Table 1:** Nutritional composition of the samples as stated on the package labeling in g∙(100 mL)^−1^.

Product	List of Ingredients	Fat	SFA ^1^	MUFA ^2^	PUFA ^3^	Protein	CHO ^4^	Energy (kcal)
Almond drink	Water, almond (7%), tapioca starch, natural almond flavoring	2.1	0.4	n/a	n/a	1	3.3	32
Coconut drink	Water, coconut milk (5.3%), raw cane sugar, maltodextrin, algae Lithothamnium Calcareum	0.9	0.9	n/a	n/a	0.1	4.1	26
Oat drink	Water, oats (10%), sea salt	0.5	0.1	n/a	n/a	1	6.5	36
Hazelnut drink	Water, sugar, hazelnuts (2.5%), calcium (tri-calcium phosphate), sea salt, stabilizers (locust bean gum, gellan gum), emulsifier (sunflower lecithin), vitamins (riboflavin B2, B12, E, D2)	1.6	0.2	1.3	0.1	0.4	3.1	29
Hemp drink	Water, hemp seeds (3%), hemp oil (1.3%), tapioca starch, emulsifier: sunflower lecithin	2.9	0.3	n/a	n/a	1	2.2	40
Quinoa drink	Water, quinoa (4%), inulin (agave fiber), sunflower oil, emulsifier: sunflower lecithin	1.2	0.1	n/a	n/a	0.5	3.5	29
Rice drink	Water, rice (11%), sunflower oil, sea salt	1.1	0.2	0.2	0.7	0.1	11	54
Soy drink	Water, shelled soybean (7.2%)	2.1	0.4	0.4	1.3	3.7	0.1	35
Cow’s milk	Whole milk 3.5% fat	3.5	2.2	n/a	n/a	3.4	4.6	63

^1^ SFA: saturated fatty acids; ^2^ MUFA: monounsaturated fatty acids; ^3^ PUFA: polyunsaturated fatty acids; ^4^ CHO: carbohydrates, n/a: not available.

**Table 2:** Fatty acid profiles of commercial plant-based milk alternatives (g/100 g total fatty acids by product). Fatty acid profile of full fat cow’s milk (3.5% fat) has been included from the Danish National Food Institute [53].

Fatty Acids	Almond Drink	Hazelnut Drink	Hemp Drink	Oat Drink	Quinoa Drink	Rice Drink	Soy Drink	Coconut Drink	Cow’s Milk
C4:0	n.d	n.d	n.d	n.d	n.d	n.d	n.d	n.d	3.91
C6:0	n.d	n.d	n.d	n.d	n.d	n.d	n.d	1.09 ± 0.10	2.64
C8:0	n.d	n.d	n.d	n.d	n.d	n.d	n.d	5.50 ± 0.07	1.46
C10:0	n.d	n.d	n.d	n.d	n.d	n.d	n.d	8.85 ± 0.21	3.11
C12:0	0.19 ± 0.03	n.d	n.d	n.d	n.d	n.d	n.d	47.20 ± 0.38	3.82
C14:0	n.d	n.d	n.d	n.d	n.d	n.d	n.d	19.37 ± 0.19	11.2
C14:1	n.d	n.d	n.d	n.d	n.d	n.d	n.d	n.d	1.03
C15:0	n.d	n.d	n.d	n.d	n.d	n.d	n.d	n.d	1.13
C16:0	13.24 ± 0.05 ^b^	5.92 ± 0.03 ^f^	6.71 ± 0.00 ^e^	17.15 ± 0.19 ^a^	6.62 ± 0.24 ^e^	8.26 ± 0.02 ^d^	9.97 ± 0.01 ^c^	8.16 ± 0.19	30.5
C16:1	0.55 ± 0.00	0.14 ± 0.00	n.d	n.d	n.d	n.d	n.d	n.d	1.65
C18:0	9.09 ± 0.06 ^a^	2.57±0.04 ^d^	3.08 ±0.02 ^c^	1.73 ± 0.05 ^f^	2.05 ± 0.00 ^e^	3.00 ± 0.01 ^c^	4.43 ± 0.00 ^b^	3.43 ± 0.27	10.5
C18:1 cis n-9	58.59 ± 0.21 ^c^	79.62 ± 0.09 ^a^	13.24 ± 0.04 ^g^	36.35 ± 0.30 ^d^	64.85 ± 1.22 ^b^	32.67 ± 0.09 ^e^	22.63 ± 0.22 ^f^	5.43 ± 0.08	20.2
C18: 1 trans-9	n.d	n.d	n.d	n.d	n.d	n.d	n.d	n.d	0.94
C18:1 trans-11	n.d	n.d	n.d	n.d	n.d	n.d	n.d	n.d	3.25
C18:2 ω-6	18.12 ± 0.07 ^e^	11.73 ± 0.01 ^f^	56.62 ± 0.04 ^a^	42.13 ± 0.17 ^c^	23.21 ± 1.09 ^d^	55.30 ± 0.13 ^b^	55.62 ± 0.14 ^ab^	0.94 ± 0.01	1.46
C20:0	n.d	n.d	2.21 ± 0.01	n.d	n.d	n.d	n.d	n.d	n.d
C18:3 ω-3	0.14 ± 0.01 ^e^	n.d	16.50 ± 0.04 ^a^	1.71 ± 0.01 ^c^	1.20 ± 0.02 ^d^	n.d	7.40 ± 0.06 ^b^	n.d	0.43
C18:3 ω-6	n.d	n.d	0.61 ± 0.00	n.d	n.d	n.d	n.d	n.d	n.d
C20:1	n.d	n.d	0.70 ± 0.02 ^b^	0.91 ± 0.01 ^a^	0.63 ± 0.01 ^c^	n.d	n.d	n.d	n.d
C22:0	n.d	n.d	0.32 ± 0.00 ^c^	n.d	0.87 ± 0.06 ^a^	0.76 ± 0.01 ^b^	n.d	n.d	n.d
C24:0	n.d	n.d	0.55 ± 0.02	n.d	n.d	n.d	n.d	n.d	n.d
SFA	22.64 ± 0.20 ^a^	8.50 ± 0.07 ^f^	12.32 ± 0.02 ^d^	18.88 ± 0.14 ^b^	10.1 ± 0.23 ^e^	12.03 ± 0.03 ^d^	14.34 ± 0.00 ^c^	93.62 ± 0.09	68.4
MUFA	59.14 ± 0.21 ^c^	79.76 ± 0.09 ^a^	13.94 ± 0.04 ^g^	37.26 ± 0.31 ^d^	65.48 ± 1.24 ^b^	32.66 ± 0.09 ^e^	22.64 ± 0.21 ^f^	5.43 ± 0.08	23.8
PUFA	18.21 ± 0.07 ^f^	11.73 ± 0.02 ^g^	73.73 ± 0.06 ^a^	43.85 ± 0.18 ^d^	24.42 ± 1.01 ^e^	55.30 ± 0.12 ^c^	63.02 ± 0.21 ^b^	0.94 ± 0.01	2.21
ω-6/ω-3	≈133	n.d	≈3.43	≈24.64	≈19.34	n.d	≈7.52	n.d	≈3.43

n.d: Not detected. Each different type of plant-based milk alternatives (PBMA) were analyzed in triplicates. One-way ANOVA was carried out within each type of fatty acid, followed by post hoc Tukey test to see significant differences (*p* < 0.05) between all samples except of coconut drink and cow’s milk. Different letters show the significant differences within each row (*p* < 0.05).

**Table 3:** In vitro protein digestibility (IVPD) of selected commercial plant-based milk alternatives and bovine serum albumin (BSA) ^1^.

Samples	Pepsin 1 h Digestibility (%)	Pancreatin 1 h Digestibility (%)	Total 2 h Digestibility (%)
Almond drink	4.82 ± 0.28 ^c^	20.28 ± 1.14 ^b^	25.19 ± 1.23 ^b^
Hemp drink	4.70 ± 0.46 ^c^	17.03 ± 0.81 ^c^	21.73 ± 1.17 ^c^
Oat drink	5.55 ± 0.05 ^bc^	19.39 ± 0.19 ^b^	24.95 ± 0.12 ^b^
Soy drink	5.96 ± 0.11 ^b^	14.61 ± 0.18 ^d^	20.56 ± 0.27 ^c^
Cow’s milk	5.06 ± 0.38 ^bc^	21.37 ± 0.74 ^b^	26.43 ± 0.84 ^b^
BSA	7.65 ± 0.47 ^a^	23.03 ± 0.43 ^a^	31.68 ± 0.33 ^a^

^1^ IVPD shows the digestibility values (%) after 1-h incubation with pepsin enzyme, 1-h incubation with pancreatic enzyme mixture, and the total digestibility after both stages of digestion (2 h). All samples were analyzed in triplicates. The results are displayed as mean ± SD values of free α-amino group concentrations in the samples detected by trinitrobenzene sulfonic acid (TNBS), in relation to alanine reference samples and an alanine amino acid standard solution representing 100% protein digestibility. One-way ANOVA was carried out within each digestion phase followed by Tukey’s post hoc test to see significant differences between samples. Different letters show the statistically significant differences (*p* < 0.05).

## Supplementary Materials

The following are available online at https://www.mdpi.com/2304-8158/9/12/1784/s1, Table S1: Composition of evaluated PBMA and cow’s milk from packaging label and after CHNS analysis of protein content from freeze-dried samples.
