# Supplementary material for: In Vitro Protein Digestibility and Fatty Acid Profile of Commercial Plant-Based Milk Alternatives
Source: Foods. 2020 Dec 1;9(12):1784. doi: 10.3390/foods9121784 (PMC7760957; doi:10.3390/foods9121784)
Supplement: Supplementary file 1 [file foods-09-01784-s001.pdf]

Supplementary Materials

Table S1. Composition of evaluated PBMA and cow's milk from packaging label and after CHNS analysis of protein content from freeze-dried samples.

	In 100 mL of the product		Freeze-dried (dry matter)	
Samples [1]	Protein content [2]	% Dry matter content [3]	% Nitrogen [4]	% Protein [5]
Almond-drink *(Ecomil)*	1.0	5.01	2.75	17.21
Hemp-drink *(Ecomil)*	1.0	4.35	2.71	16.94
Oat-drink *(Oatly)*	1.0	11.24	1.90	11.92
Soy-drink *(Naturli)*	3.7	6.96	8.11	50.73
Cow's milk *(Arla)*	3.4	12.25	4.35	27.81

[1] Samples selected: PBMA with the highest protein content from all the different brands and the least of additives included.

[2] The list of ingredients and the protein content was obtained from the package label. Protein content was given as g/100 mL.

[3] The percentage of dry matter content (g/100 mL) was calculated after freeze-drying the samples.

[4] The % of nitrogen presented was obtained from CHNS analysis of ≅ 30 mg of freeze-dried sample.

[5] The % of the protein was calculated with the estimated conversion factor 6.25 from nitrogen content (%) (AOAC, 2000)
